# Production and scavenging of reactive oxygen species both affect reproductive success in male and female *Drosophila melanogaster*

**DOI:** 10.1007/s10522-021-09922-1

**Published:** 2021-04-26

**Authors:** Biz R. Turnell, Luisa Kumpitsch, Klaus Reinhardt

**Affiliations:** Applied Zoology, Faculty Biology, Technische Universität Dresden, 01069 Dresden, Germany

**Keywords:** Alternative oxidase, Antioxidants, *dj-1*β, *DJ-1*, Oxygen radicals, Sperm aging

## Abstract

Sperm aging is accelerated by the buildup of reactive oxygen species (ROS), which cause oxidative damage to various cellular components. Aging can be slowed by limiting the production of mitochondrial ROS and by increasing the production of antioxidants, both of which can be generated in the sperm cell itself or in the surrounding somatic tissues of the male and female reproductive tracts. However, few studies have compared the separate contributions of ROS production and ROS scavenging to sperm aging, or to cellular aging in general. We measured reproductive fitness in two lines of *Drosophila melanogaster* genetically engineered to (1) produce fewer ROS via expression of alternative oxidase (AOX), an alternative respiratory pathway; or (2) scavenge fewer ROS due to a loss-of-function mutation in the antioxidant gene *dj-1*β. Wild-type females mated to AOX males had increased fecundity and longer fertility durations, consistent with slower aging in AOX sperm. Contrary to expectations, fitness was not reduced in wild-type females mated to *dj-1*β males. Fecundity and fertility duration were increased in AOX and decreased in *dj-1*β females, indicating that female ROS levels may affect aging rates in stored sperm and/or eggs. Finally, we found evidence that accelerated aging in *dj-1*β sperm may have selected for more frequent mating. Our results help to clarify the relative roles of ROS production and ROS scavenging in the male and female reproductive systems.

**Supplementary Information:**

The online version contains supplementary material available at 10.1007/s10522-021-09922-1.

## Introduction

Aging in sperm cells has broad implications for sexual selection (Reinhardt 2007; Pizzari et al. 2008) and reproductive aging, but has received comparatively little attention. Sperm, like all cells, are subject to the cellular aging process and the resulting accumulation of cellular damage (Reinhardt 2007; Pizzari et al. 2008). For sperm, the consequences of this aging process are especially serious, since, unlike most cells, they cannot slow tissue aging by simply blocking the cell cycle when too much damage has built up, but rather must stay functional up until the moment of fertilization.

In addition, sperm are particularly vulnerable to a major agent of age-related cellular damage, reactive oxygen species (ROS). While ROS play an important role in cellular signaling (Sohal and Orr 2012), including in sperm cells (Aitken et al. 2016), an imbalance between ROS production and ROS scavenging by antioxidants causes oxidative damage to lipids, proteins, DNA, and other cellular components. Most cellular ROS are generated by the mitochondrial electron transport chain during aerobic respiration, when electrons flowing down the chain are transferred to molecular oxygen instead of the next subunit. The resulting superoxide (O_2_^−^), and the more stable hydrogen peroxide (H_2_O_2_) to which it is converted, are neutralized by antioxidants like superoxide dismutases and peroxidases (Balaban et al. 2005).

The oxidative stress caused by ROS is especially problematic for sperm, with their limited antioxidant reserves and membranes rich in oxidation-prone polyunsaturated fatty acids (Aitken 2020). Sperm aging impairs several aspects of sperm function, including motility (Aitken 2020), velocity (Gasparini et al. 2017; Vega-Trejo et al. 2019), storage in the female (Reinhardt and Siva-Jothy 2005), and fertilization capacity (White et al. 2008; Reinhardt and Ribou 2013). Aged sperm can also accumulate oxidative damage to the DNA, decreasing offspring survival (Tarín et al. 2000; Levitas et al. 2005). These negative effects reduce the fitness of both the male providing the sperm and the female using them (White et al. 2008; Tan et al. 2013; Gasparini et al. 2017).

Sperm are exposed to ROS and antioxidants from both endogenous and exogenous sources. In species with aerobic sperm metabolism, sperm produce their own mitochondrial ROS (Ribou and Reinhardt 2012; Reinhardt and Ribou 2013; Paynter et al. 2017; Moraes and Meyers 2018; Turnell and Reinhardt 2020). Exogenous sources include the male reproductive organs where sperm are produced and stored prior to mating and the female reproductive tract where they are stored prior to fertilization. In males, ROS are generated in mammals by leukocytes in the semen (Sikka 2012) and by Sertoli cells in the seminiferous tubules (Du Plessis et al. 2015). Antioxidants are found in the mammalian epididymis (Jervis and Robaire 2004) and, across a range of vertebrate and invertebrate taxa, in the seminal fluid (Poiani 2006; Baer et al. 2009b), which has been called one of the most powerfully antioxidative substances on Earth (Wathes et al. 2007).

Similarly, the female reproductive tract also generates both ROS (Rizzo et al. 2012; Agarwal et al. 2017) and antioxidants (Heifetz and Rivlin 2010). In many insects, antioxidant genes are upregulated in the sperm storage organs of mated females (Baer et al. 2009a; Prokupek et al. 2009; Shaw et al. 2014; Gonzalez et al. 2018). Females can thus limit the aging rate of stored sperm by providing antioxidants, by decreasing their own ROS production, and/or by decreasing the ROS production of the sperm themselves (Paynter et al. 2017; Reinhardt and Ribou 2013; Ribou and Reinhardt 2012). In addition to these physiological responses, females may also employ behavioral ones to avoid the costs of using aged sperm, for example by mating frequently to refresh their sperm stores (Reinhardt 2007).

While oxidative stress levels are determined by both ROS production and ROS scavenging, few studies have directly compared the separate contributions of these two mechanisms to the sperm aging process. Two genetic models have been particularly useful in determining the respective physiological effects of ROS production and ROS scavenging: AOX and *DJ-1*. AOX, or alternative oxidase, is an electron transport chain protein, absent in vertebrates and arthropods but found in many other taxa (McDonald et al. 2009), that decreases ROS formation by bypassing the cytochrome chain (Amirsadeghi et al. 2006). AOX expression has been shown to reduce ROS production in transgenic *Drosophila melanogaster* (Fernández-Ayala et al. 2009; Sanz et al. 2010) and mice (El-Khoury et al. 2013).

Sperm from AOX-expressing males are predicted to experience a lower rate of cellular damage accumulation during both male and female storage, leading to an improved and longer-lasting fertilization capability. Previously, we found that sperm from *D. melanogaster* males with somatic AOX expression did not itself generate less ROS than sperm from control males (Turnell et al. 2021). As *D. melanogaster* sperm have been shown to undergo oxidative phosphorylation and generate ROS (Turnell and Reinhardt 2020), this result suggests that the promoter used (daGal4) does not drive expression in the germline. However, as sperm are subject to environmental as well as endogenous ROS, they are predicted to suffer less oxidative stress when produced and stored by males and females expressing AOX in the somatic tissues of the reproductive organs.

*DJ-1* is a gene whose mutation increases oxidative stress in humans and animals (Canet-Avilés et al. 2004; Kim et al. 2005). In *Drosophila*, loss of function of the homologous gene *dj-1*β leads to increased H_2_O_2_ production and oxidative stress (Andres-Mateos et al. 2007; Fernández-Ayala et al. 2009; Sanz et al. 2010; Stefanatos et al. 2012; Saari et al. 2017), as well as locomotory defects (Park et al. 2005). The dj-1*β* protein acts as ROS scavenger itself (Taira et al. 2004; Andres-Mateos et al. 2007) and can also promote the function of other antioxidants through transcriptional regulation (Van Der Brug et al. 2008; Blackinton et al. 2009) and enzyme activation (Wang et al. 2011; Girotto et al. 2014).

*DJ-1* has been shown to affect sperm function in a range of animals. In humans, sperm with lower concentrations of the DJ-1 protein show reduced sperm motility and increased oxidative stress (An et al. 2011; Sun et al. 2014; Nowicka-Bauer et al. 2018). DJ-1 levels are correlated with sperm membrane integrity and/or normal sperm morphology in rams (Favareto et al. 2010) and bulls (Boe-Hansen et al. 2015). In rats, both sperm motility and antioxidant activity decrease concomitantly with DJ-1 levels over the male’s lifetime (Takemura et al. 2014), while exposure to fertility-reducing toxins reduces sperm DJ-1 concentrations in mice (Okada et al. 2002) and rabbits (Veeramachaneni et al. 2007). Just as AOX expression may theoretically decrease ROS production both by the sperm itself (given successful germline expression) and by the male and female storage organs, a loss of function of *dj-1*β may decrease antioxidant production both in sperm and in the surrounding environment.

Here, we tested the hypothesis, based on the theoretical expectations and the empirical evidence presented above, that the expression of AOX and the loss of function of *dj-1*β respectively cause decelerated and accelerated aging in sperm, leading to corresponding increases or decreases in reproductive fitness. As explained above, this aging may be exacerbated by ROS, and ameliorated by antioxidants, stemming both from the sperm itself and from the male and female somatic tissues. We compared fecundity and fertility duration in wild-type Canton-S (hereafter referred to as “CS”) *D. melanogaster* females mated to males expressing either the AOX gene (“AOX” males) or a knockdown mutation of the *dj-1*β gene (“*dj-1*β” males), as well as to wild-type control males with same genetic background as the transgenic and mutant lines (Dahomey, “DAH”). We also performed the reverse experiment, mating AOX, *dj-1*β, and DAH control females to wild-type CS males, in order to detect any effects on sperm function mediated by decreased somatic ROS production or scavenging in the female sperm storage organs.

Because female responses to sperm aging may include more frequent mating, as noted above, we also measured the remating rates of CS females receiving AOX, *dj-1*β, or DAH control sperm; and AOX, *dj-1*β, and DAH control females receiving CS sperm. These experiments tested the following predictions: (1) Females can proximately detect sperm aging and remate accordingly (that is, less frequently for AOX females or females receiving AOX sperm, and more frequently for *dj-1*β females or females receiving *dj-1*β sperm); (2) *dj-1*β females are under selection to minimize the costs of sperm aging through more frequent mating. Our results demonstrate the important consequences of ROS production and ROS scavenging on male and female reproductive fitness.

## Methods

### Fly lines and husbandry

UAS-AOX F6 (Fernández-Ayala et al. 2009), *daughterless*-Gal4 driver (daGal4) (Wodarz et al. 1995) (previously BL55849), *dj-1*β^*GE23381*^ (Park et al. 2005), and *white* wild-type Dahomey (Broughton et al. 2005) flies were kindly provided by Dr. Alberto Sanz (then University of Newcastle). The AOX, daGal4, and *dj-1*β lines had been backcrossed into the DAH background for 11, 11, and 7 generations, respectively (Sanz et al. 2010). In the current study, AOX-expressing AOX/daGal4 flies were generated by mating AOX males to virgin daGal4 females. Flies were maintained at 25 °C and 60% humidity on a 12:12 light:dark cycle and fed on a yeast-corn-sugar medium (40 g/l yeast, 90 g/l corn meal, 100 g/l sucrose, 12 g/l agar, 40 ml/l nipagin [10% in ethanol] and 3 ml/l propionic acid in water). Males and females were separated upon eclosion and kept in groups of approximately eight individuals in 25 mm vials until use. Females were provided with additional yeast during this time.

### Mating observations

AOX/daGal4, *dj-1*β, and DAH control males/females were paired with wild-type CS females/males, either once (single matings) or repeatedly over the course of 10 days (ad libitum matings), for a total of four experiments. Individual virgin females (3–4 days post-eclosion) were placed with single novel males (4–6 days post-eclosion) in 25 mm vials containing standard yeast-corn-sugar medium at approximately 09:00 and observed continuously until approximately 16:00. For the single mating experiments, females that did not mate on the 1st day were given a second opportunity to mate on the following day, except where otherwise noted. All males were virgin except in the male effects ad libitum mating experiment, in which a subset of females in each group were presented each day with a new male that had previously been given one opportunity to mate but had then had at least 2 days to recover. There was no difference across groups in the proportion of females paired with these possible nonvirgin mates (DAH: 34/62; AOX/daGal4: 39/65; *dj-1*β: 42/69; *χ*^2^ = 0.8, *p* = 0.68). Male mating history had no effect on female mating frequency, mating latency, fecundity, or fertility duration (all *p* > 0.05).

### Fecundity, fertility duration, and lifespan

For the single mating experiments, females were transferred to fresh vials every day for the first 10 days following a successful mating. Eggs were counted and vials were maintained under the environmental conditions described above. For each female, the total number of adult offspring emerging these ten vials was divided by the total number of eggs laid to calculate egg-to-adult success. After 10 days, females were transferred to fresh vials every 3–4 days for the duration of their lifespans, as were females in the ad libitum mating experiments. For all experiments, the adult offspring emerging in each vial were removed and counted approximately 12 days after the start of oviposition, with a second count approximately 3 days later to ensure that all emergent adults had been recorded. Fertility duration was defined as the number of days post-mating that a female continued to produce eggs that would develop into adult offspring. Female survival was noted twice weekly for all females until their natural deaths, except in the female effects ad libitum mating experiment, where survival was monitored for 40 days.

### Seminal vesicle and accessory gland area

Because AOX-expressing AOX/daGal4 flies resulted from a cross between two populations, they may have been subject to less potential for inbreeding depression than *dj-1*β or DAH control flies. To separate the effects of AOX expression and outbreeding on male reproductive fitness, AOX-nonexpressing, outbred flies were generated by mating AOX males to virgin DAH females. These AOX/DAH flies carried the AOX gene but not the daGal4 driver necessary for AOX expression. As a proxy measure for general male reproductive fitness, seminal vesicle and accessory gland areas were measured in AOX/daGal4, AOX/DAH, *dj-1*β, and DAH control males. See Supplementary Information for detailed methods.

### Statistical analysis

Likelihoods of mating and of producing offspring were compared using Chi-squared tests with post-hoc pairwise comparisons via Fisher’s exact tests (for these and all other post-hoc comparisons, *p*-values were adjusted using the Holm method). Females given only one opportunity to mate in the single mating experiments were excluded from the mating likelihood analyses. Females producing no offspring were excluded from all other analyses. Mating frequency, mating latency, egg and adult offspring production, and egg-to-adult offspring survival were compared across groups using Kruskal–Wallis tests with post-hoc pairwise comparisons via Dunn’s tests.

Fertility duration and lifespan were compared via log-ranks tests with post-hoc pairwise comparisons using the R package survival (Therneau 2020). Adult offspring production over time was compared across lines with regression analysis using the R package gamlss (Rigby and Stasinopoulos 2005), via a zero-inflated negative binomial model with line and the number of days post-mating as fixed effects and female ID as a random effect.

Effect sizes were calculated for all Kruskal–Wallis and ANOVA (*η*^2^) and Chi-squared (Cohen’s *w*) tests (Cohen 1992; Morris and Fritz 2013). These are marked in Tables 1, 2, 3, and 4 with * for a moderate effect (*w* ≈ 0.3, η^2^ ≈ 0.06) or ** for a large effect (Cohen’s *w* ≈ 0.5, η^2^ ≈ 0.14). *p*-values in these tables are reported for the overall test as well as for the post-hoc comparisons between the control and AOX and between the control and *dj-1*β. Bold text in these tables indicates a significant difference in AOX or *dj-1*β compared to the DAH control group. Summary statistics are reported as mean ± sd, with [n] reported as necessary. All statistics were performed in R version 3.6.1 (R Core Team 2020). Results are summarized in Tables 5 and 6.

**Table 1:** Mating behavior results for CS females mated singly or ad libitum over 10 days to AOX/daGal4, *dj-1*β, and DAH control males

	Mating behavior	Male line			χ^2^	Effect size	*p*	*p* vs. AOX	*p* vs. *dj-1*β
		DAH	AOX	*dj-1*β					
Single matings	Females mated	81/94 (86%)	93/100 (93%)	**56/98 (57%)**	42.59	0.38*	< 0.0001	0.16	< 0.0001
	Mated on day 1	62/81 (77%)	78/93 (84%)	**33/56 (59%)**	11.78	0.20	0.003	0.25	0.003
*Ad lib* matings	Mated multiply	35/62 (56%)	**50/63 (79%)**	46/69 (67%)	7.52	0.20	0.023	0.022	0.28
	# Matings	1.76 ± 0.80	**2.30 ± 0.96**	1.88 ± 0.80	12.41	0.05	0.002	0.002	0.34
	1st mating latency	2.11 ± 1.76	**1.38 ± 0.68**	2.32 ± 1.79	19.83	0.09*	< 0.0005	0.006	0.18
	Avg remating latency	4.25 ± 2.41	**2.72 ± 1.88**	3.69 ± 2.09	11.84	0.08*	0.003	0.010	0.83

Statistically significant differences from the DAH control group are shown in bold; moderate and large effect sizes are marked with one or two asterisks, respectively. Averages are mean ± SD

**Table 2:** Mating behavior results for AOX/daGal4, *dj-1*β, and DAH control females mated singly or ad libitum over 10 days to CS males

	Mating behavior	Female line						*p* vs. AOX	*p* vs. *dj-1*β
		DAH	AOX	*dj-1*β	χ^2^	Effect size	*p*		
Single matings	Females mated	52/71 (73%)	55/84 (65%)	**71/81 (88%)**	11.20	0.22	0.004	0.38	0.003
	Mated on day 1	43/52 (82%)	35/55 (63%)	67/71 (94%)	19.45	0.33*	< 0.0001	0.06	0.07
Ad lib matings	Mated multiply	32/58 (55%)	36/72 (50%)	41/60 (68%)	4.66	0.16	0.10	0.60	0.37
	# Matings	1.62 ± 0.62	1.58 ± 0.64	**2.00 ± 0.88**	9.28	0.04	0.010	0.70	0.037
	1st mating latency	1.14 ± 0.35	**1.01 ± 0.12**	**1.03 ± 0.18**	9.98	0.04	0.007	0.008	0.031
	Avg remating latency	2.58 ± 2.18	3.89 ± 2.95	**4.08 ± 2.42**	8.94	0.07*	0.011	0.10	0.009

Statistically significant differences from the DAH control group are shown in bold; moderate and large effect sizes are marked with one or two asterisks, respectively. Averages are mean ± SD

**Table 3:** Fitness results for CS females mated singly or ad libitum over 10 days to AOX/daGal4, *dj-1*β, and DAH control males

	Fitness measure	Male line			χ^2^	Effect size	*p*	*p* vs. AOX	*p* vs. *dj-1*β
		DAH	AOX	*dj-1*β					
Single matings	Females w/offspring	77/82 (93%)	**70/93 (75%)**	**86/105 (82%)**	11.04	0.20	0.004	0.002	0.032
	# Eggs, 10 days	90 ± 28	96 ± 35	83 ± 31	7.62	0.02	0.022	0.27	0.21
	Egg-to-adult survival	0.87 ± 0.12	0.89 ± 0.13	0.85 ± 0.14	10.51	0.04	0.005	0.13	0.16
	# Offspring, 10 days	79 ± 26	**87 ± 34**	72 ± 29	12.74	0.05	0.002	0.048	0.21
	# Offspring, lifetime	89 ± 31	**142 ± 63**	82 ± 36	53.38	0.22**	< 0.0001	< 0.0001	0.21
	Days fertile	12.3 ± 6.7	**25.0 ± 9.9**	11.7 ± 5.6	85.1	0.37**	< 0.0001	< 0.0001	0.38
	Lifespan (days)	59.2 ± 10.2	**60.7 ± 11.1**	57.8 ± 11.7	16.6	0.04	< 0.001	0.003	0.53
Ad lib matings	Females w/offspring	62/62 (100%)	63/65 (97%)	69/69 (100%)	4.07	0.14	0.13	0.99	1.00
	# Offspring, lifetime	346 ± 96	380 ± 96	378 ± 97	2.99	0.01	0.22	0.42	0.28
	Days fertile	18.3 ± 6.9	**23.9 ± 6.9**	19.2 ± 5.8	21.5	0.09*	< 0.0001	0.0001	0.62
	Lifespan (days)	65.1 ± 10.6 [57]	68.0 ± 10.3 [52]	66.8 ± 11.3 [64]	3.2	0.01	0.20	0.28	0.43

“Offspring” and “Days fertile” refer to the production of adult offspring. Statistically significant differences from the DAH control group are shown in bold; moderate and large effect sizes are marked with one or two asterisks, respectively. Averages are mean ± SD, with [n] shown if less than the total sample size

**Table 4:** Fitness results for AOX/daGal4, *dj-1*β, and DAH control females mated singly or ad libitum over 10 days to CS males

	Fitness measure	Female line						*p* vs. AOX	*p* vs. *dj-1*β
		DAH	AOX	*dj-1*β	χ^2^	Effect size	*p*		
Single matings	Females w/offspring	62/67 (93%)	66/68 (97%)	65/76 (86%)	6.26	0.17	0.044	0.55	0.55
	# Eggs, 10 days	100 ± 28	95 ± 29	**84 ± 34**	6.55	0.03	0.038	0.32	0.037
	Egg-to-adult survival	0.57 ± 0.18	**0.81 ± 0.14**	**0.31 ± 0.17**	115.3	0.65**	< 0.0001	< 0.0001	< 0.0001
	# Offspring, 10 days	57 ± 25	**77 ± 29**	**28 ± 21**	75.93	0.42**	< 0.0001	0.004	< 0.0001
	# Offspring, lifetime	84 ± 44 [47]	99 ± 36 [66]	**33 ± 24 [65]**	80.66	0.45**	< 0.0001	0.054	< 0.0001
	Days fertile	19.4 ± 6.1 [47]	**16.8 ± 3.9** [66]	**12.0 ± 5.7 [65]**	42.9	0.19**	< 0.0001	< 0.001	< 0.0001
	Lifespan (days)	53.6 ± 10.9 [34]	52.7 ± 10.6 [42]	**63.0 ± 19.6 [42]**	17.2	0.03	< 0.001	0.75	0.004
Ad lib matings	Females w/offspring	58/59 (98%)	72/74 (97%)	60/65 (92%)	3.41	0.13	0.18	1	0.63
	# Offspring, lifetime	82 ± 38	**108 ± 35**	**45 ± 39**	64.90	0.34**	< 0.0001	< 0.001	< 0.0001
	Days fertile	13.9 ± 6.1	15.6 ± 6.1	12.2 ± 5.8	9.1	0.03	0.010	0.25	0.25
	Survival to 40 days	33/58 (57%)	49/72 (68%)	**17/60 (28%)**	21.46	0.33*	< 0.0001	0.20	0.005
	Lifespan, < 40 days	32.3 ± 2.7 [25]	33.6 ± 3.0 [23]	30.4 ± 3.9 [43]	11.4	0.05	0.003	0.20	0.10

“Offspring” and “Days fertile” refer to the production of adult offspring. Statistically significant differences from the DAH control group are shown in bold; moderate and large effect sizes are marked with one or two asterisks, respectively. Averages are mean ± SD, with [n] shown if less than the total sample size

**Table 5:** Regression results of male line and days post-mating on the production of adult offspring by CS females mated singly or ad libitum over 10 days to AOX/daGal4, *dj-1*β, and DAH control males (zero-inflated negative binomial model with female ID as a random effect)

	Male effect	Estimate (s.e.)	*t*	*p*
Single matings	Intercept	2.566 (0.127)	20.245	< 0.0001
	AOX/daGal4	0.240 (0.214)	1.123	0.26
	***dj-1β***	− 0.323 (0.156)	− 2.063	0.039
	**Days post-mating**	− 0.213 (0.008)	− 27.189	< 0.0001
	**AOX/daGal4 × Days**	0.090 (0.011)	8.333	< 0.0001
	***dj-1β × Days***	0.022 (0.009)	2.490	0.013
Ad lib matings	Intercept	7.373 (0.300)	24.582	< 0.0001
	AOX	− 0.002 (0.397)	− 0.004	1
	*dj-1*β	0.612 (0.393)	1.554	0.12
	**Days post-mating**	− 0.338 (0.012)	− 28.650	< 0.0001
	**AOX × Days**	0.066 (0.014)	4.593	< 0.0001
	*dj-1*β × Days	− 0.016 (0.015)	− 1.032	0.30

Statistically significant effects are shown in bold

**Table 6:** Regression results of female line and days post-mating on the production of adult offspring by AOX/daGal4, *dj-1*β, and DAH control females mated singly or ad libitum over 10 days to CS males (zero-inflated negative binomial model with female ID as a random effect)

	Female effect	Estimate (s.e.)	*t*	*p*
Single matings	Intercept	2.264 (0.092)	24.618	< 0.0001
	**AOX**	0.561 (0.118)	4.747	< 0.0001
	***dj-1β***	− 0.678 (0.122)	− 5.543	< 0.0001
	**Days post-mating**	− 0.072 (0.006)	− 12.198	< 0.0001
	**AOX × Days**	− 0.043 (0.008)	− 5.241	< 0.0001
	***dj-1β × Days***	− 0.063 (0.009)	− 6.687	< 0.0001
*Ad lib* matings	Intercept	5.074 (0.116)	43.875	< 0.0001
	AOX	0.263 (0.151)	1.743	0.0815
	***dj-1β***	− 0.928 (0.170)	− 5.467	< 0.0001
	**Days post-mating**	− 0.339 (0.011)	− 29.656	< 0.0001
	AOX × Days	0.018 (0.014)	1.249	0.21
	*dj-1*β × Days	− 0.001 (0.017)	− 0.052	0.96

Statistically significant effects are shown in bold

## Results

### Part I: mating behavior

#### Male effects (CS females)

*Single matings:* Females paired with *dj-1*β males were 34% less likely to mate and 23% less likely to mate at the first opportunity, compared to females paired with DAH control males (Table 1). Females paired with AOX/daGal4 males did not differ from the control group in any mating latency measures.

Ad libitum *matings:* Females paired with AOX/daGal4 males mated 31% more often (Fig. 4a, Table 1) and were 41% more likely to mate multiply, compared to females paired with DAH control males. These females also had a 35% shorter latency to the first mating and a 36% shorter average remating latency. Females paired with *dj-1*β males did not differ from the control group in any mating frequency or latency measures.

#### Female effects (CS males)

*Single matings: dj-1*β females were 21% more likely to mate than DAH control females (Table 2). Among females that did mate, *dj-1*β females also tended to be more likely to mate at the first opportunity, while AOX/daGal4 females tended to be less likely to do so.

Ad libitum *matings: dj-1*β females mated 23% more often than DAH control females (Fig. 4b, Table 2). There was no difference across groups in the incidence of multiple mating. *dj-1*β females had a 9% shorter latency to first mating but a 58% longer average remating latency than control females. AOX/daGal4 females likewise had an 11% shorter first mating latency and tended to have a longer average remating latency, by 51%, compared to control females.

### Part II: fitness measures

#### Male effects (CS females)

*Single matings:* Females paired with *dj-1*β males were 12% less likely to produce adult offspring, compared to females paired with DAH control males (Table 3). Females paired with AOX/daGal4 males were 19% less likely to produce adult offspring. During the first 10 days after mating, females mated to AOX/daGal4 males produced 10% more adult offspring compared to controls. There were no differences among groups in the number of eggs laid during the first 10 days or in egg-to-adult offspring survival.

**Fig. 1:**
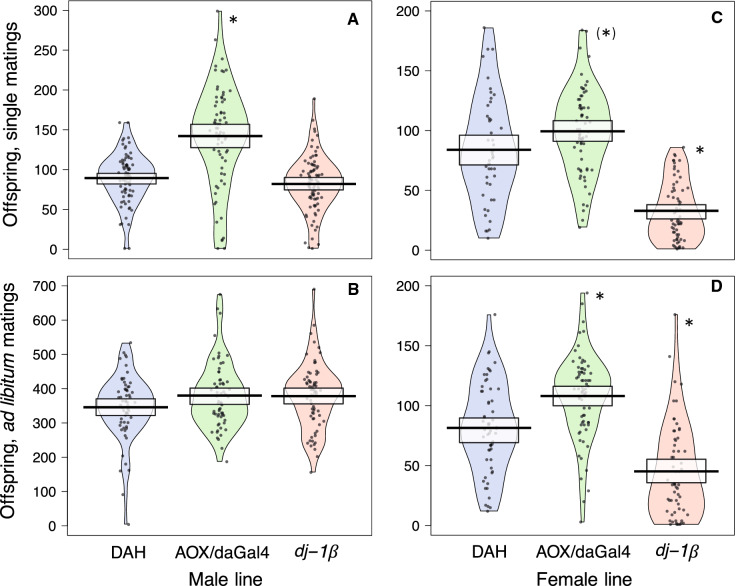
Total lifetime adult offspring production. CS females were mated to AOX/daGal4, *dj-1*β, and DAH control males (**a**, **b**) and AOX/daGal4, *dj-1*β, and DAH control females were mated to CS males (**c**, **d**). Females were mated once (**a**, **c**) or ad libitum over the course of 10 days (**b**, **d**). Asterisks indicate a significant difference from the DAH control (Kruskal–Wallis test with post-hoc pairwise comparisons; asterisk in parentheses indicates a trend, *p* < 0.1). Black line = mean, white box = 95% CI. Note the different y-axis scales

**Fig. 2:**
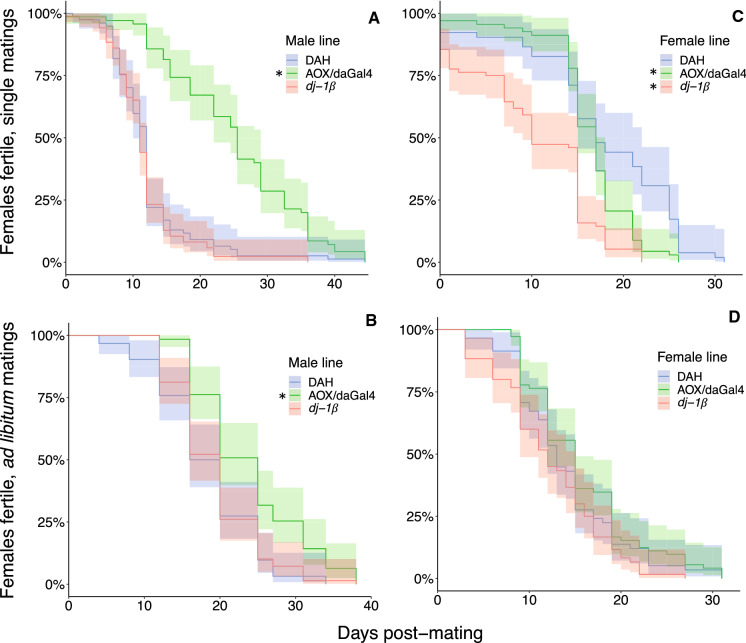
Percentage of females producing adult offspring over time. CS females were mated to AOX/daGal4, *dj-1*β, and DAH control males (**a**, **b**) and AOX/daGal4, *dj-1*β, and DAH control females were mated to CS males (**c**, **d**). Females were mated once (**a**, **c**) or ad libitum over the course of 10 days (**b**, **d**). Asterisks indicate a significant difference from the DAH control (log-ranks tests with post-hoc pairwise comparisons). Shading shows 95% CI. Note the different x-axis scales

**Fig. 3:**
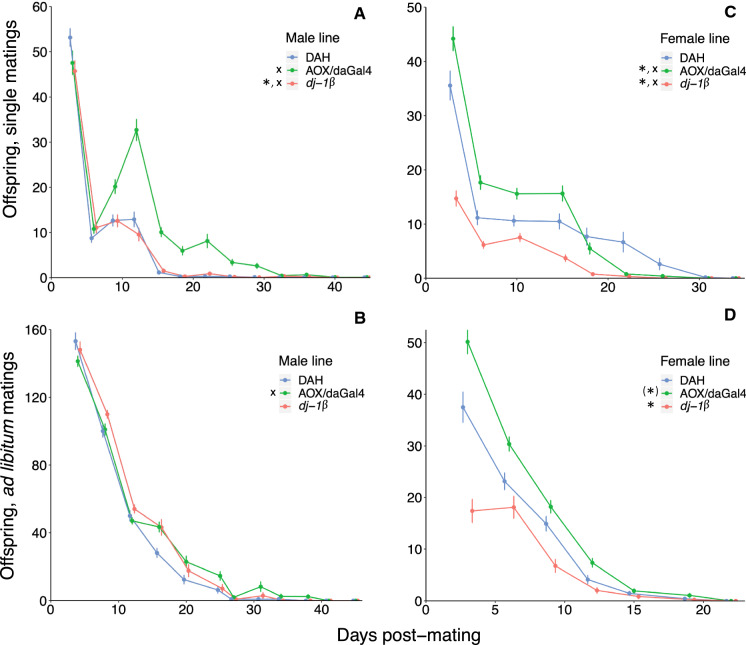
Production over time of adult offspring. CS females were mated to AOX/daGal4, *dj-1*β, and DAH control males (**a**, **b**) and AOX/daGal4, *dj-1*β, and DAH control females were mated to CS males (**c**, **d**). Females were mated once (**a**, **c**) or ad libitum over the course of 10 days (**b**, **d**). Asterisks indicate a significant main effect of line vs. the DAH control (zero-inflated negative binomial regression; asterisk in parentheses indicates a trend, *p* < 0.1); x’s indicate a significant interaction between line and time. Error bars indicate s.e. Note the different axes scales

Over the course of their lifetimes, females mated to AOX/daGal4 males produced 59% more adult offspring compared to controls (Fig. 1a) and were fertile for twice as long (Fig. 2a), despite living only slightly longer. Females mated to *dj-1*β males did not differ from the DAH control group in any of the above measures. In the regression analysis, both females mated to AOX/daGal4 and to *dj-1*β males had slower declines over time in adult offspring production compared to controls (Table 7, Fig. 3a). Females mated to *dj-1*β males also had lower overall adult offspring production in the regression analysis.

**Table 7:** Summary of mating behavior and fitness results for CS females mated to AOX/daGal4, *dj-1*β, and DAH control males and whether they support (**✓**) or refute (X) the sperm aging hypothesis and the hypothesis that females proximately detect stored sperm aging and remate accordingly

	AOX males	Sperm aging?	Decreased remating?
Mating behavior	Equally likely to mate (single)		
	Equally likely to mate on 1st day (single)		
	More likely to mate multiply (ad lib)		X
	More frequent mating (ad lib)		X
	Shorter first mating latency (ad lib)		
	Shorter remating latency (ad lib)		X
Fitness, single matings	Less likely to produce adult offspring	X	
	Equal egg number, first 10 days		
	Equal egg-to-adult offspring survival		
	More adult offspring, first 10 days	**✓**	
	More adult offspring, lifetime	**✓**	
	Longer fertility duration	**✓**	
	Slower decline in offspring production	**✓**	
Fitness, ad lib matings	Equally likely to produce adult offspring		
	Equal lifetime adult offspring number		
	Longer fertility duration	**✓**	
	Slower decline in offspring production	**✓**	

	*dj-1*β males	Sperm aging?	Increased remating?
Mating behavior	Less likely to mate (single)		
	Less likely to mate on 1st day (single)		
	Equally likely to mate multiply (ad lib)		
	Equal mating frequency (ad lib)		
	Shorter first mating latency (ad lib)		
	Shorter remating latency (ad lib)		**✓**
Fitness, single matings	Less likely to produce adult offspring	**✓**	
	Equal egg number, first 10 days		
	Equal egg-to-adult offspring survival		
	Equal offspring number, first 10 days		
	Fewer adult offspring, lifetime^†^	**✓**	
	Equal fertility duration		
	Slower decline in offspring production	X	
Fitness, ad lib matings	Equally likely to produce adult offspring		
	Equal offspring number, lifetime		
	Longer fertility duration	X	
	Slower decline in offspring production	X	

“Offspring production” and “fertility duration” refer to the production of adult offspring

^†^GLMM results

*A*d libitum *matings:* The groups did not differ in the total number of adult offspring produced over the course of their lifetimes (Table 3, Fig. 1b). Females mated to AOX/daGal4 males had a 31% longer fertility duration (Fig. 2b) and, in the regression analysis, a slower decline in adult offspring production over time (Table 7, Fig. 3b) compared to controls. Females mated to *dj-1*β males did not differ from controls in any fecundity or fertility duration measures. Lifespan did not differ across groups.

#### Female effects (CS males)

*Single matings:* During the first 10 days after mating, AOX/daGal4 females produced 35% more adult offspring and *dj-1*β females produced 51% fewer adult offspring compared to DAH control females (Table 4). *dj-1*β females also laid 16% fewer eggs during this period. Egg-to-adult offspring survival was 42% higher in AOX/daGal4 females and 46% lower in *dj-1*β females.

Over the course of their lifetimes, *dj-1*β females produced 61% fewer adult offspring and AOX/daGal4 females tended to produce more (by 18%) compared to control females (Fig. 1c). AOX/daGal4 and *dj-1*β females had shorter fertility durations than DAH control females, by 13% and 38%, respectively (Fig. 2c). In the regression analysis, AOX/daGal4 females had higher overall adult offspring production but a faster decline in offspring production over time compared to controls, while *dj-1*β females had both lower overall adult offspring production and a faster decline in adult offspring production over time (Table 8, Fig. 3c). Lifespan was 18% longer in *dj-1*β females.

**Table 8:** Summary of mating behavior and fitness results for AOX/daGal4, *dj-1*β, and DAH control females mated to CS males and whether they support (**✓**) or refute (X) the sperm aging hypothesis and the hypotheses that (1) females proximately detect stored sperm aging and remate accordingly (*dj-1*β females and CS females mated to *dj-1*β males) and (2) *dj-1*β females have been selected for increased remating

	AOX females	Sperm aging?	Decreased remating?
Mating behavior	Equally likely to mate (single)		
	Equally likely to mate on 1st day (single)		
	Equally likely to mate multiply (ad lib)		
	Equal mating frequency (ad lib)		
	Shorter first mating latency (ad lib)		
	Equal remating latency (ad lib)		
Fitness, single matings	Equally likely to produce adult offspring		
	Equal egg number, first 10 days		
	Higher egg-to-adult offspring survival	**✓**	
	More adult offspring, first ten days	**✓**	
	More adult offspring, lifetime^†^	**✓**	
	Shorter fertility duration	X	
	Faster decline in offspring production	X	
Fintess, ad lib matings	Equally likely to produce adult offspring		
	More adult offspring, lifetime	**✓**	
	Equal fertility duration		
	Equal decline in offspring production		

	*dj-1*β females	Sperm aging?	Increased remating?
Mating behavior	More likely to mate (single)		**✓**
	Equally likely to mate on 1st day (single)		
	Equally likely to mate multiply (ad lib)		
	More frequent mating (ad lib)	**✓**	**✓**
	Shorter first mating latency (ad lib)	**✓**	**✓**
	Longer remating latency (ad lib)	X	X
Fitness, single matings	Equally likely to produce adult offspring		
	Fewer eggs laid, first 10 days	**✓**	
	Lower egg-to-adult offspring survival	**✓**	
	Fewer offspring, first 10 days	**✓**	
	Fewer offspring, lifetime	**✓**	
	Shorter fertility duration	**✓**	
	Faster decline in offspring production	**✓**	
Fitness, ad lib matings	Equally likely to produce adult offspring		
	Fewer offspring, lifetime	**✓**	
	Shorter fertility duration	**✓**	
	Equal decline in offspring production		

“Offspring production” and “fertility duration” refer to the production of adult offspring

^†^GLMM results

Ad libitum *matings:* AOX/daGal4 females produced 33% more and *dj-1*β females produced 45% fewer adult offspring over the course of their lifetimes than control females (Table 4, Fig. 1d). Fertility duration was 12% shorter in *dj-1*β females (Fig. 2d). In the regression analysis, AOX/daGal4 females tended to have higher overall adult offspring production while *dj-1*β females had lower overall offspring production, but the decline in adult offspring production over time did not differ across groups (Table 8, Fig. 3d). *dj-1*β females were 49% less likely than controls to survive to 40 days.

#### Seminal vesicle and accessory gland area

AOX/DAH males had larger accessory glands than all other lines and larger seminal vesicles than AOX/daGal4 males. We therefore found no evidence for heterosis in AOX/daGal4 flies. See Supplementary Information for detailed results and discussion.

## Discussion

We separated the fitness effects of decreased ROS production, here represented by the alternative mitochondrial pathway AOX model, and decreased ROS scavenging, here represented by the loss-of-function mutant *dj-1*β model. Our results show that the reproductive fitness of wild-type males and females is limited by their ability to minimize ROS production on the one hand and maximize ROS scavenging on the other. Females had higher fecundity and a longer duration of fertility both when mated to males with decreased ROS production and when they themselves produced fewer ROS. Conversely, fecundity was lower and the duration of fertility shorter in females that scavenged fewer ROS. These findings support the hypothesis that reactive oxygen species levels influence the aging rate of sperm in the male and the female storage environments, with major consequences for male and female fitness.

### Increased fecundity and fertility duration in females mated to AOX/daGal4 males, and in AOX/daGal4 females, suggest that wild-type fitness is limited by male and female ROS production

Females mated singly to AOX/daGal4 males had nearly 60% more offspring (Fig. 1a), a doubly long fertility duration (Fig. 2a), and a slower decline over time in offspring production (Fig. 3a) compared to females mated to DAH control males. Females mated ad libitum to AOX/daGal4 males also had higher fecundity (Fig. 1b) and a slower fecundity decline (Fig. 3b), though they did not differ in fertility duration (Fig. 2b). These results support the hypothesis that AOX/daGal4 sperm is subject to less oxidative stress, resulting in slower aging. Because the rate of sperm ROS production is not reduced in AOX/daGal4 males compared to DAH control males (the authors, in review), this decelerated aging is likely due to the lower levels of exogenous ROS experienced by sperm during spermatogenesis and storage in the testes and seminal vesicles of AOX/daGal4 males, tissues in which AOX is successfully expressed (Saari et al. 2017).

Like wild-type females mated to AOX/daGal4 males, AOX/daGal4 females mated to wild-type males also had higher fitness than control females, producing more offspring (when mated ad libitum, Fig. 1d) or tending to do so (when mated once, Fig. 1c). They also had higher rates of egg-to-adult offspring survival and a longer fertility duration than control females when mated once (Fig. 2c). These results suggest that sperm senescence can be delayed by lower ROS levels in the female sperm storage environment. While the faster decline in offspring production over time shown by singly-mated AOX/daGal4 females (Fig. 3c) does not fit this prediction, this decline may simply be due to their higher initial fecundity.

Several other factors may also have contributed to the high fecundity of AOX/daGal4 females. First, decreased ROS levels may have prevented oxidative damage not only in the AOX/daGal4 females’ sperm stores, but also in their eggs. While sperm, for reasons outlined above, are particularly vulnerable to oxidative stress, eggs are also susceptible (Lord and John Aitken 2013; Perkins et al. 2016; Aitken 2020). On the other hand, there is evidence in *D. melanogaster* that ovaries are actually more resistant than somatic tissue to oxidative stress (Tsakiri et al. 2013). In addition, eggs in *D. melanogaster* are continuously produced (Bastock and St Johnston 2008), and, in contrast to the sperm that is stored by females for several weeks after mating, are thus likely to spend only a short time in storage before being used. Nevertheless, further work is necessary to determine the extent to which egg aging impacts reproductive fitness.

A second alternative explanation for the AOX/daGal4 females’ high fecundity is that AOX expression may have increased these females’ attractiveness, causing their mates to provide them with more sperm and/or seminal fluid proteins via strategic ejaculate tailoring (Lüpold et al. 2011; Kelly and Jennions 2011; Hopkins et al. 2019). Any fitness effect of somatic ROS production in the AOX/daGal4 females’ offspring themselves can be ruled out: because these offspring lacked either the AOX gene or the daGal4 driver, they did not express AOX.

### Decreased fecundity and fertility duration in *dj-1*β females suggest that wild-type fitness is enhanced by ROS scavenging

*dj-1*β females had fewer offspring (Fig. 1c, d) and a lower rate of egg-to-adult survival than control females; singly-mated *dj-1*β females also had a shorter fertility duration (Fig. 2c) and a faster decline in offspring production over time (Fig. 3c). These results are consistent with the hypothesis that sperm stored by *dj-1*β females is subject to more oxidative stress, resulting in faster aging. This elevated stress could result from both (1) increased attack from unneutralized female-generated ROS and (2) decreased availability of female-generated antioxidants that could neutralize ROS generated by the sperm. In *D. melanogaster*, a number of antioxidant genes are upregulated in the female sperm storage organs after mating (Mack et al. 2006; Prokupek et al. 2009; reviewed in Heifetz and Rivlin 2010). Indeed, these organs show high antioxidant levels in a wide range of taxa, from mammals and birds (reviewed in Holt and Fazeli 2016) to social insects (Baer et al. 2009a; Gotoh et al. 2017), evidencing the critical role females play in preserving stored sperm.

As with the elevated fecundity of AOX/daGal4 females, the reduced fecundity of *dj-1*β females may also have been caused by decreased antioxidant activity in their eggs and/or by the receipt of smaller ejaculates due to lower attractiveness. Their low rate of egg-to-adult offspring survival could also theoretically be due to decreased antioxidant production in the heterozygous mutant offspring themselves, if the effects of the *dj-1*β gene are dose-dependent (e.g., Yang et al. 2005). This explanation seems unlikely, however, since survival was not decreased in the offspring of *dj-1*β males.

As with endogenous ROS production in AOX/daGal4 sperm, we found no evidence that endogenous ROS scavenging differed in *dj-1*β compared to control sperm. Females mated to *dj-1*β males did not show reductions in total offspring production (Fig. 1a, b), egg-to-adult offspring survival, or fertility duration (Fig. 2a, b), nor, when mated ad libitum, did they have a faster decline in offspring production over time (Fig. 3b). [While the regression analysis did find a negative main effect on offspring production in females mated singly to *dj-1*β males, this was accompanied by a slower decline over time (Table 2, Fig. 3a)]. These results suggest that *dj-1*β sperm are not subject to increased oxidative stress due to reduced scavenging activity.

That *dj-1*β sperm themselves would not perform less ROS scavenging is unsurprising: because of their low cytoplasmic volume, sperm in general have very limited antioxidant capabilities (Aitken et al. 2016). More surprising is that the environment of the *dj-1*β male reproductive system, where sperm are presumably subject to higher ROS levels, did not affect sperm fitness. It is possible that the testes and seminal vesicles provide only negligible amounts of antioxidants to sperm during spermatogenesis and storage (though see Mahfouz et al. 2009 for contradictory evidence in humans). Alternatively, the severity of the oxidative stress caused by limited somatic antioxidant availability may be relatively mild in at least some sperm cells, such that enough sufficiently functional sperm are transferred during mating to ensure normal female fecundity and fertility. In this case, *dj-1*β males may be expected to show reduced postcopulatory fitness only under conditions of sperm competition, if their sperm are capable of fertilization but are outperformed by sperm from non-mutant males.

### Increased mating frequency in *dj-1*β females suggests that ROS scavenging may allow wild-type females to minimize the costs of mating

**Fig. 4:**
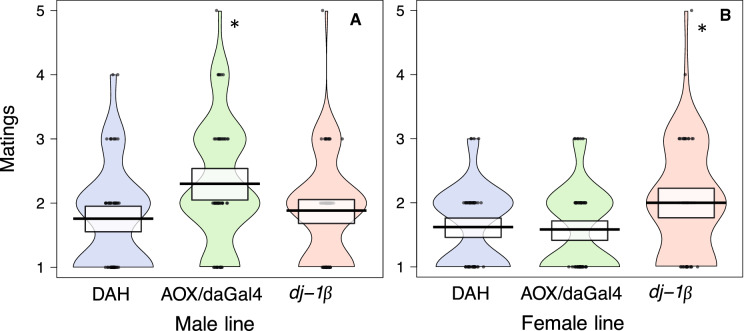
Number of matings over the course of 10 consecutive days. **a** CS females × AOX/daGal4, *dj-1*β, and DAH control males; **b** AOX/daGal4, *dj-1*β, and DAH control females × CS males. Females were provided each day with a novel virgin male. Asterisks indicate a significant difference from the DAH control (Kruskal–Wallis test with post-hoc pairwise comparisons). Black line = mean, white box = 95% CI

In addition to having reduced fecundity and a shorter fertility duration, *dj-1*β females also mated more often than control females (Fig. 4b). This finding supports the hypothesis that rapid sperm senescence can select for frequent female remating. Evidence for such selection has been found across species of *Drosophila*: females mate more often in species with quickly metabolizing sperm, where cellular damage is expected to accumulate faster (the authors, in review). By supplying their sperm stores with antioxidants, females may slow the process of sperm aging, allowing them to rely on a single ejaculate for a longer period of time and thus to minimize costly remating (Arnqvist and Nilsson 2000; Wigby and Chapman 2005; Kuijper et al. 2006).

If low sperm ROS levels reduce female mating rate, then AOX/daGal4 females may be expected to mate less often than wild-type females. The AOX/daGal4 females used in this study cannot have been under selection for a decreased mating rate, however, since they were created from two separate populations, AOX and daGal4, neither of which should differ from control flies in their ROS levels. While it is possible that AOX/daGal4 females, or females mated to AOX/daGal4 males, may nevertheless be able to detect the slower rate of aging in their sperm stores and may accordingly mate less often, we found no evidence for such a plastic response. Indeed, wild-type females actually mated more often when paired with AOX/daGal4 males than with control males, suggesting that AOX/daGal4 males are more attractive due to AOX expression.

In conclusion, we found substantial differences in male and female reproductive fitness in otherwise genetically identical *D. melanogaster* lines differing in their rates of ROS production or scavenging. While further work may be needed to tease apart aging in eggs and aging in female-stored sperm, our findings in males indicate, and our findings in females suggest, that sperm aging plays an important evolutionary role. The fitness consequences of sperm aging via oxidative stress may drive selection on not only cellular traits like ROS production and scavenging, but also organismal traits like mating frequency. Mating rates, in turn, have implications not only for sexual selection but also for population genetics, conservation, epidemiology, and a range of other fields (Pizzari and Wedell 2013; Taylor et al. 2014).

Sperm aging may also drive the evolution of population-level traits. For example, sexual conflict over sperm metabolic rate may arise if rapid metabolism gives males an edge during sperm competition but also causes accelerated aging in female storage. Such a pattern has been shown in rodents, where quickly metabolizing sperm swim faster and are therefore more competitive (Tourmente et al. 2013, 2019), but also suffer increased DNA fragmentation (DelBarco-Trillo et al. 2016). These evolutionary implications underscore the significance of sperm aging via oxidative stress as a powerful selective force.

## Supplementary Information

Below is the link to the electronic supplementary material.

Supplementary file1 (DOCX 21 kb)

Supplementary file2 (PDF 205 kb)

Supplementary file3 (PDF 119 kb)

## Data Availability

Data are archived at Zenodo (10.5281/zenodo.4679710).
