# Supplementary material for: Production and scavenging of reactive oxygen species both affect reproductive success in male and female Drosophila melanogaster
Source: Biogerontology. 2021 Apr 26;22(4):379–96. doi: 10.1007/s10522-021-09922-1 (PMC8266701; doi:10.1007/s10522-021-09922-1)
Supplement: Supplementary file 1 — Supplementary file1 (DOCX 21 kb) [file 10522_2021_9922_MOESM1_ESM.docx]

**Supplementary information**

**Methods**

Seminal vesicle and accessory gland areas were measured in AOX/daGal4, *dj-1*β, DAH, and AOX-nonexpressing AOX/DAH flies. Glands from 14-day-old virgin males were dissected in PBS and photographed using a Leica DFC9000 camera on a DMi8 microscope. The areas of one seminal vesicle and one accessory gland were measured for each male in ImageJ (NIH). Accessory gland areas were compared using ANOVAs with post-hoc pairwise comparisons via Tukey’s tests. Seminal vesicle areas were compared using Kruskal-Wallis tests with post-hoc pairwise comparisons via Dunn’s tests. Effect sizes (*η*^2^) are marked with * for a moderate effect (𝜂^2^ ≈ 0.06) or ** for a large effect (𝜂^2^ ≈ 0.14).

**Results**

*Seminal vesicle and accessory gland area*

Seminal vesicle gland size differed significantly across lines (Fig. S1, Table S1; 𝜒^2^ = 14.03, *η*^2^ = 0.18**, *p* = 0.003), being 29% larger in AOX/DAH than in AOX/daGal4 males. Accessory glands were larger in AOX/DAH males than in all other lines (Fig. S2, Table S1; *F* = 8.31, *η*^2^ = 0.33**, *p* = 0.0001; AOX/DAH vs. DAH, 21% larger; vs. AOX/daGal4, 30% larger; vs. *dj-1*β, 29% larger).

**Discussion**

*Alternative explanation: reduced inbreeding depression in AOX/daGal4 flies?*

Although the DAH, AOX, daGal4, and *dj-1*β flies were outbred, it is still possible that deleterious recessive mutations may have accumulated in these lines. The negative fitness effects of such mutations would likely be stronger in DAH and *dj-1*β flies than in AOX/daGal4 flies, which were the product of a cross between two populations. To investigate this possibility, we measured two indicators of male reproductive fitness: seminal vesicle size, which is correlated with sperm number; and accessory gland size, which is correlated with the quantity of seminal fluid proteins (Wigby et al. 2009). Transferring more sperm may increase female fecundity or fertility duration via decreased sperm limitation, while transferring more seminal fluid proteins may do so via oviposition stimulation or enhanced sperm uptake, storage, and usage (Ram and Wolfner 2007; Sirot et al. 2015).

We found no evidence for heterosis in AOX/daGal4 flies. Seminal vesicles and accessory glands were no larger in AOX/daGal4 than in *dj-1*β or DAH control males, being if anything relatively small (Figure S1; Table S1). The increased fecundity and fertility durations of females mated to AOX/daGal4 males can therefore not be attributed to the receipt of more sperm or seminal fluid proteins. Interestingly, we did find evidence for heterosis in AOX-nonexpressing, outbred AOX/DAH males, which had larger accessory glands than all other lines (Figure S2; Table S1). Why a positive effect of outbreeding on gland size would be seen in AOX/DAH but not AOX/daGal4 males is unclear, unless this effect is somehow offset by the expression of AOX. AOX/daGal4 flies show slight increases in development time and post-eclosion weight loss, possibly because the AOX pathway produces not only less ROS but also less ATP (Fernández-Ayala et al. 2009).

**References**

Fernández-Ayala DJM, Sanz A, Vartiainen S, et al (2009) Expression of the *Ciona intestinalis* alternative oxidase (AOX) in *Drosophila* complements defects in mitochondrial oxidative phosphorylation. Cell Metab 9:449–460. https://doi.org/10.1016/j.cmet.2009.03.004

Ram KR, Wolfner MF (2007) Seminal influences: *Drosophila* Acps and the molecular interplay between males and females during reproduction. Integr Comp Biol 47:427–445. https://doi.org/10.1093/icb/icm046

Sirot LK, Wong A, Chapman T, Wolfner MF (2015) Sexual conflict and seminal fluid proteins: A dynamic landscape of sexual interactions. Cold Spring Harb Perspect Biol 7:a017533. https://doi.org/10.1101/cshperspect.a017533

Wigby S, Sirot LK, Linklater JR, et al (2009) Seminal fluid protein allocation and male reproductive success. Curr Biol 19:751–757. https://doi.org/10.1016/j.cub.2009.03.036

**Table S1: Comparisons of seminal vesicle and accessory gland area across the different lines.** Summary statistics are reported as mean ± sd [n]. See text for details of the statistical analysis.

| **Seminal vesicle** | **Area (mm^2^)** | ***p* vs. AOX/DAH** |
| --- | --- | --- |
| **DAH** | 0.042 ± 0.008 [15] | 0.30 |
| **AOX/daGal4** | 0.035 ± 0.008 [16] | 0.001 |
| **AOX/DAH** | 0.045 ± 0.004 [15] | - |
| ***dj-1*β** | 0.041 ± 0.005 [12] | 0.20 |
| **Accessory gland** |  |  |
| **DAH** | 0.171 ± 0.026 [13] | 0.014 |
| **AOX/daGal4** | 0.158 ± 0.032 [17] | < 0.0005 |
| **AOX/DAH** | 0.206 ± 0.025 [13] | - |
| ***dj-1*β** | 0.159 ± 0.029 [11] | 0.001 |

**Figure S1: Seminal vesicle area in AOX/daGal4, *dj-1*β, DAH control, and AOX-nonexpressing AOX/DAH males.** Asterisk indicates a significant difference between two groups (ANOVA with post-hoc pairwise comparisons). Black line = mean, white box = 95% CI.

**Figure S2: Accessory gland area in AOX/daGal4, *dj-1*β, DAH control, and AOX-nonexpressing AOX/DAH males.** Asterisk indicates a significant difference from all other groups (Kruskal-Wallis test with post-hoc pairwise comparisons). Black line = mean, white box = 95% CI.
