# Supplementary figures and images for: Production and scavenging of reactive oxygen species both affect reproductive success in male and female Drosophila melanogaster
Source: Biogerontology. 2021 Apr 26;22(4):379–96. doi: 10.1007/s10522-021-09922-1 (PMC8266701; doi:10.1007/s10522-021-09922-1)

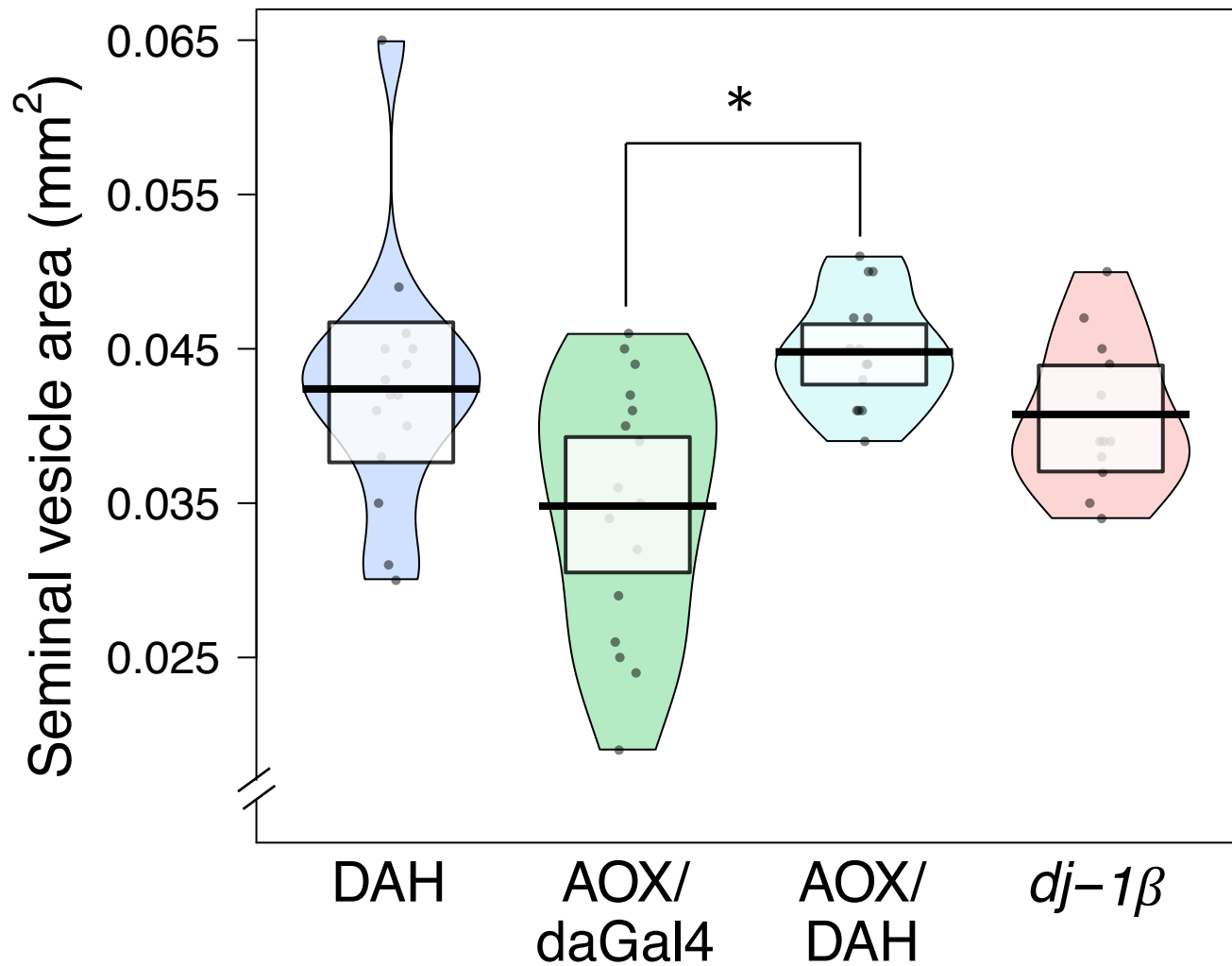

Supplement: Supplementary file 2 — Supplementary file2 (PDF 205 kb) [file 10522_2021_9922_MOESM2_ESM.pdf]

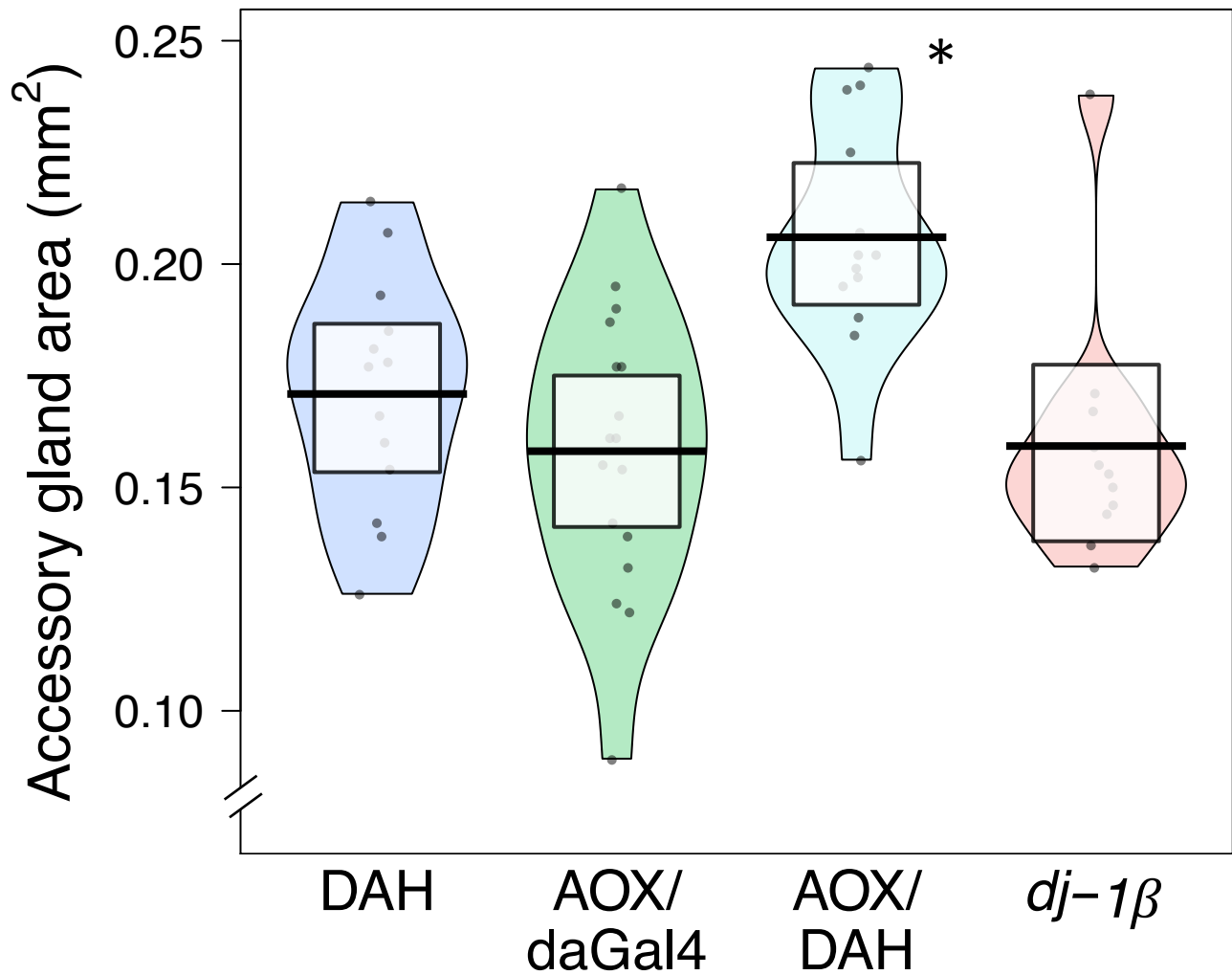

Supplement: Supplementary file 3 — Supplementary file3 (PDF 119 kb) [file 10522_2021_9922_MOESM3_ESM.pdf]
